# Small area estimation of under-5 mortality in Bangladesh, Cameroon, Chad, Mozambique, Uganda, and Zambia using spatially misaligned data

**DOI:** 10.1186/s12963-018-0171-7

**Published:** 2018-08-13

**Authors:** Laura Dwyer-Lindgren, Ellen R. Squires, Stephanie Teeple, Gloria Ikilezi, D. Allen Roberts, Danny V. Colombara, Sarah Katherine Allen, Stanley M. Kamande, Nicholas Graetz, Abraham D. Flaxman, Charbel El Bcheraoui, Kristjana Asbjornsdottir, Gilbert Asiimwe, Ângelo Augusto, Orvalho Augusto, Baltazar Chilundo, Caroline De Schacht, Sarah Gimbel, Carol Kamya, Faith Namugaya, Felix Masiye, Cremildo Mauieia, Yodé Miangotar, Honoré Mimche, Acácio Sabonete, Haribondhu Sarma, Kenneth Sherr, Moses Simuyemba, Aaron Chisha Sinyangwe, Jasim Uddin, Bradley H. Wagenaar, Stephen S. Lim

**Affiliations:** 1 Institute for Health Metrics and Evaluation, University of Washington, 2301 5th Ave, Suite 600, Seattle, WA 98103 USA; 2 Health Alliance International, Seattle, WA USA; 3 Infectious Disease Research Collaboration, Kampala, Uganda; 4 Instituto Nacional da Saúde, Maputo, Mozambique; 5 Department of Community Medicine, Eduardo Mondlane University, Maputo, Mozambique; 6 Health Alliance International, Maputo, Mozambique; 7 Department of Family & Child Nursing, University of Washington, Seattle, WA USA; 8 University of Zambia, Lusaka, Zambia; 9 University of N’Djamena, N’Djamena, Chad; 10 Institut de Formation et de Recherche Démographiques, University of Yaoundé II, Yaoundé, Cameroon; 11 International Centre for Diarrhoeal Disease Research, Dhaka, Bangladesh; 12 Department of Global Health, University of Washington, Seattle, WA USA

**Keywords:** U5MR, Small area estimation, Subnational, Geographic disparities, Inequalities

## Abstract

**Background:**

The under-5 mortality rate (U5MR) is an important metric of child health and survival. Country-level estimates of U5MR are readily available, but efforts to estimate U5MR subnationally have been limited, in part, due to spatial misalignment of available data sources (e.g., use of different administrative levels, or as a result of historical boundary changes).

**Methods:**

We analyzed all available complete and summary birth history data in surveys and censuses in six countries (Bangladesh, Cameroon, Chad, Mozambique, Uganda, and Zambia) at the finest geographic level available in each data source. We then developed small area estimation models capable of incorporating spatially misaligned data. These small area estimation models were applied to the birth history data in order to estimate trends in U5MR from 1980 to 2015 at the second administrative level in Cameroon, Chad, Mozambique, Uganda, and Zambia and at the third administrative level in Bangladesh.

**Results:**

We found substantial variation in U5MR in all six countries: there was more than a two-fold difference in U5MR between the area with the highest rate and the area with the lowest rate in every country. All areas in all countries experienced declines in U5MR between 1980 and 2015, but the degree varied both within and between countries. In Cameroon, Chad, Mozambique, and Zambia we found areas with U5MRs in 2015 that were higher than in other parts of the same country in 1980. Comparing subnational U5MR to country-level targets for the Millennium Development Goals (MDG), we find that 12.8% of areas in Bangladesh did not meet the country-level target, although the country as whole did. A minority of areas in Chad, Mozambique, Uganda, and Zambia met the country-level MDG targets while these countries as a whole did not.

**Conclusions:**

Subnational estimates of U5MR reveal significant within-country variation. These estimates could be used for identifying high-need areas and positive deviants, tracking trends in geographic inequalities, and evaluating progress towards international development targets such as the Sustainable Development Goals.

**Electronic supplementary material:**

The online version of this article (10.1186/s12963-018-0171-7) contains supplementary material, which is available to authorized users.

## Background

A country’s under-5 mortality rate (U5MR) – the probability that a child will die before reaching his or her fifth birthday – is a widely used measure of health and development. The Millennium Development Goals [1] (MDGs) and, more recently, the Sustainable Development Goals [2] (SDGs) both include targets related to child mortality: for the MDGs, a two-thirds reduction in U5MR between 1990 and 2015, and for the SDGs a reduction in U5MR to less than 25 deaths per 1000 live births by 2030. Country-level estimates of U5MR are available globally from a number of sources [3, 4]; however, detailed subnational estimates (e.g., at the second administrative level) are not widely available. This is unfortunate, as subnational estimates are essential for measuring inequalities and are important for public health planning and evaluation purposes. Furthermore, subnational estimates could be used in monitoring progress towards development goals such as the SDGs and MDGs to ensure that certain geographic regions are not systematically left behind.

In developing countries, U5MR is usually estimated on the basis of birth history data collected in surveys and censuses, wherein women are interviewed about their children’s survival. Methods for analyzing these data have been available for decades [5, 6] and have been widely applied at the country level. However, technical challenges have prevented their widespread use for generating subnational estimates. In particular, the number of child deaths in any given survey within a limited geographic area is generally quite small and estimates based on a small number of events are subject to random fluctuations.

In recent years, researchers have begun to apply small area estimation methods – statistical methods specifically designed to deal with the issues posed by small numbers – to birth history data in order to estimate subnational U5MR [7–10]. These methods take advantage of spatial and temporal correlation in U5MR and, where appropriate, smooth estimates across adjacent areas and time periods. These methods often allow for combining multiple data sources, which can partially alleviate the small numbers problem, and facilitate analyzing trends over longer periods of time.

However, a major limitation of most existing small area estimation methods is that they are only able to make use of data sources that identify the precise geographies of interest. Data which are ‘spatially misaligned’ compared to the geographies of interest – e.g., data for higher administrative units or for historical boundaries no longer in effect – have typically been discarded. In many cases, a large proportion of the available data fall in this category, forcing researchers to choose between analyzing at a more aggregate level or using only a fraction of the available data. For example, due to frequent changes in district boundaries in Uganda, researchers have previously chosen to analyze U5MR and other maternal and child health indicators at the much coarser region level rather than the district level [9].

A recent analysis by Golding et al. [11] utilized model-based geostatistics methods to estimate gridded surfaces of U5MR in Africa using point data (i.e., data associated with GPS coordinates). This method allows for incorporation of data from arbitrary areas, including spatially misaligned data, by re-assigning these data to a series of point locations proportional to population within the given area. While this approach allows for utilizing spatially misaligned data, it essentially assumes that the same U5MR was observed in all locations within a given area described in the spatially misaligned data. This assumption is likely unrealistic in some cases, particularly for larger areas (e.g., provinces or regions).

For this analysis, we developed small area estimation methods that are sufficiently flexible to incorporate data for any geography that can be formed by combining one or more of the areas of interest, allowing data at multiple geographic levels to be incorporated while still estimating for the finest geographic level. We then applied this method to all available data in Bangladesh, Cameroon, Chad, Mozambique, Uganda, and Zambia in order to generate annual subnational estimates of U5MR at the second (and in the case of Bangladesh, third) administrative level from 1980 to 2015. To demonstrate the utility of geographically precise information on U5MR, we use these estimates to explore spatial and temporal patterns in U5MR, quantify within-country inequalities, and evaluate subnational progress towards meeting MDG and SDG targets.

## Methods

### Unit of analysis

We estimated U5MR at the second administrative level in Cameroon (departments), Chad (departments), Mozambique (districts), Uganda (districts), and Zambia (districts); and at the third administrative level in Bangladesh (sub-districts). Table 1 describes the administrative hierarchy for each country.

**Table 1:** Administrative units

Country	Admin. level 1	Admin. level 2	Admin. level 3
Bangladesh	Division (7)	District (64)	Sub-district (484)^a^
Cameroon	Region (10)	Department (58)	–
Chad	Region (23)^b^	Department (62)^b, c^	–
Mozambique	Province (11)^d^	District (148)^d^	–
Uganda	Region (4)	District (112)	–
Zambia	Province (9)^e^	District (72)^e^	–

^a^Some highly urbanized areas of Bangladesh are classified as city corporations which we treat as equivalent to sub-districts for this analysis

^b^In Chad, the capital city is considered equivalent to both a region and a department

^c^Chad currently has 68 departments. However, the most detailed data source identified used the 62 departments in effect until 2011, so we have carried out the analysis at this level

^d^In Mozambique, the capital city is considered equivalent to a province while the provincial capital cities are considered equivalent to districts

^e^Zambia currently has 10 provinces and 103 districts. However, district boundary changes in recent years have not been well documented. We therefore carry out this analysis on the 9 provinces and 72 districts that were in effect until approximately 2010

### Population counts

Annual under-5 population estimates were compiled for each unit of analysis in each country from 1980 to 2015 from a combination of census microdata, census reports, and existing population series (Table 2) with the primary goal of describing the relative population size of different areas within a country rather than the precise absolute population size in any one area. For Bangladesh, Mozambique, Uganda, and Zambia, we sought age-specific census microdata or tabulations at the finest geographic level available. Where the appropriate level of geographic detail was not available – either because census counts were tabulated at a higher level (e.g., province or region) or because administrative boundary definitions had changed over time – the counts were split according to the proportions observed in the next census year. When only total (all ages combined) tabulations were available, these were age-split according to the age patterns observed in the closest census year with age-specific data available in the same country. For Mozambique, we supplemented the available census data with an existing district-level population series available via the Spatial Data Repository [12]. Total and age-specific populations for each area were interpolated and projected from 1980 to 2015 assuming geometric growth and then age-specific populations were scaled to match the total population in each year in each area. For Cameroon and Chad, where less age- and area-specific census data were readily available, we instead utilized the gridded population estimates from WorldPop [13, 14]. We overlaid the population grid on a current shapefile and then aggregated the population within each area to generate department-level population estimates for each country. WorldPop estimates were available for 2000, 2005, 2010, and 2015, and we utilized these four sets of estimates for 1980–2000, 2001–2005, 2006–2010, and 2011–2015, respectively, holding the population constant within each time period.

**Table 2:** Population data sources

Years	Geographic level	Age detail	Source
Bangladesh			
1974, 1981	National	Total	Census tabulation
1991, 2001	Upazila^a^	Age-specific	Census tabulation
2011	Upazila	Age-specific	Census tabulation
Cameroon			
2000, 2005, 2010, 2015	1-km grid	Age-specific	WorldPop
Chad			
2000, 2005, 2010, 2015	1-km grid	Age-specific	WorldPop
Mozambique			
1980	Province	Age-specific	Census tabulation
2000–2015	District	Age-specific	Spatial Data Repository
Uganda			
1980, 1991, 2002	District	Total	Census tabulation
1991, 2002	District^a^	Age-specific	Census microdata
2014	District	Age-specific	Census tabulation
Zambia			
1990	District^a^	Age-specific	Census microdata
2000, 2010	District	Age-specific	Census microdata

^a^Historical administrative boundary sets which require splitting to match current administrative boundaries

### Birth history data

For this analysis, we identified surveys and censuses in all six countries which contained summary birth history (SBH) or complete birth history (CBH) data (Table 3). For surveys that contained the latitude and longitude of each survey cluster, we overlaid these coordinates with a current district, department, or sub-district (depending on the country) shape file and identified the area each survey cluster belonged to. In all other surveys, as well as in censuses, we extracted the finest geographic level identified in the available data. Birth history data for each geographic area included in a given survey or census were analyzed independently using the complete or summary birth history methods described below. When both complete and summary birth history data were available from a single survey, only complete birth history data were analyzed and included in the analysis.

**Table 3:** Birth history data sources

Survey^a^	Geographic level^b^	Spatially aligned?^c^	Complete birth history	Summary birth history
Bangladesh				
1993–1994 DHS	District (64)	No	x	
1996–1997 DHS	District (64)	No	x	
1999–2000 DHS	Sub-district (484)	Yes	x	
2001 DHS	District (64)	No	x	
2004 DHS	Sub-district (484)	Yes	x	
2007 DHS	Sub-district (484)	Yes	x	
2010 MMHCS	District (64)	No	x	
2011–2012 DHS	Sub-district (484)	Yes	x	
2012–2013 MICS	District (64)	No		x
2014 DHS	Sub-district (484)	Yes	x	
Cameroon				
1991 DHS	Department (58)	Yes	x	
1998 DHS	Region (10)	No	x	
2000 MICS	Region (10)	No		x
2004 DHS	Department (58)	Yes	x	
2011 DHS	Department (58)	Yes	x	
2014 MICS	Region (10)	No	x	
Chad^d^				
1996–1997 DHS	Region (15)	No	x	
2000 MICS	Region (15)	No		x
2004 DHS	DHS Region (9)	No	x	
2010 MICS	Department (62)	Yes		x
2014–2015 DHS	Department (62)	Yes	x	
Mozambique				
1997 Census	District (146)	No		x
1997 DHS	Province (11)	No	x	
2003 DHS	Province (11)	No	x	
2007 Census	District (148)	Yes		x
2008 MICS	Province (11)	No	x	
2009 AIS	District (148)	Yes		x
2011 DHS	District (148)	Yes	x	
Uganda				
1991 Census	District (38)	No		x
1992–1993 UNIHS	District (38)	No		x
1995 DHS	District (38)	No	x	
2000–2001 DHS	District (112)	Yes	x	
2002 Census	District (56)	No		x
2006 DHS	District (112)	Yes	x	
2009–2010 MIS	District (112)	Yes	x	
2009–2010 UNPS	District (87)	No		x
2010–2011 UNPS	District (112)	Yes		x
2011 AIS	District (112)	Yes		x
2011 DHS	District (112)	Yes	x	
2011–2012 UNPS	District (112)	Yes		x
2014–2015 MIS	District (112)	Yes		x
Zambia				
1990 Census	District (57)	No		x
1992 DHS	District (57)	No	x	
1996–1997 DHS	District (57)	No	x	
2000 Census	District (72)	Yes		x
2001–2002 DHS	Province (9)	No	x	
2007 DHS	District (72)	Yes	x	
2010 Census	District (72)	Yes		x
2013–2014 DHS	District (72)	Yes	x	

^a^*AIS* AIDS Indicator Survey, *DHS* Demographic and Health Survey, *MICS* Multiple Indicator Cluster Survey, *MMHCS* Maternal Mortality and Healthcare Survey, *MIS* Malaria Indicator Survey, *UNIHS* Uganda National Integrated Household Survey, *UNPS* Uganda National Panel Survey

^b^Numbers shown in parentheses indicate the number of areas in a given set of administrative boundaries. This is to distinguish between current and historical sets of areas that go by the same name

^c^Sources that could be analyzed at the current second administrative level (third, in Bangladesh only), as described in Table 1, are considered spatially aligned while all other data sources are considered spatially misaligned

^d^Chad previously had prefectures which are roughly equivalent to regions. For simplicity, both regions and prefectures are listed as regions in this table. ‘DHS region’ are an alternate set of regions defined for statistical purposes in the 2004 Demographic and Health Survey

### Analysis of complete birth histories

We extracted child-level data on date of birth, survival status, age at death (if applicable), and sampling weights from surveys with complete birth histories. The data for each child were expanded to encompass each month that the child started alive prior to reaching age 5 or the date of the survey, whichever occurred first. All child-months were subdivided by calendar year and into six age groups (month 0, months 1–11, year 1, year 2, year 3, and year 4) and the monthly probability of death in each age-year group was calculated as the weighted proportion of child months which ended in death, where the weights were the survey sample weights. We then derived U5MR for each calendar year as:

$$ U5{MR}_{j,t,s}^{(CBH)}=1-{\prod}_a{\left(1-{q}_{j,t,s,a}\right)}^{n_a} $$

where *q*_*j*, *t*, *s*, *a*_ is the monthly probability of death in area *j*, year *t*, age group *a,* source *s*; and *n*_*a*_ is the number of months in age group *a*.

We estimated ${N}_{j,t,s}^{(CBH)}$, the number of births associated with a given $U5{MR}_{j,t,s}^{(CBH)}$ estimate based on the number of child months contributing to that estimate. Specifically, the number of child months in area *j*, year *t*, and source *s* (summed across the six age groups) was divided by the mean number of months children in source *s* lived prior to death, reaching age 5, or the time of survey, whichever occurred first. We then estimated ${Y}_{j,t,s}^{(CBH)}$, the number of deaths associated with each U5MR estimate, by multiplying the estimated number of births by the estimated U5MR:

$$ {Y}_{j,t,s}^{(CBH)}=U5M{R}_{j,t,s}^{(CBH)}\cdotp {N}_{j,t,s}^{(CBH)} $$

### Analysis of summary birth histories

We extracted woman-level data on the number of children ever born and the number of children died from surveys and censuses with summary birth histories. We then applied the combined summary birth history method described by Rajaratnam et al. [15] in order to generate preliminary estimates of U5MR by area and year from each survey. This method requires several inputs, indexed by mother’s age or reported time since first birth, including: number of women, total children ever born, total children died, and mean children born (per woman). Women-level sample weights were used to generate weighted estimates of all input parameters. The output of these summary birth history methods are annual estimates of U5MR for approximately 25 years preceding the date of the survey or census.

As an intermediate step in applying the summary birth history methods described by Rajaratnam et al. [15], all reported births are distributed on an annual basis to the years preceding the survey or census using empirical distributions of time since birth indexed by mother’s age and the reported number of children at the time of survey. The resulting annual estimates of births (${N}_{j,t,s}^{\ast (SBH)}$) were taken as the starting point for estimating an effective sample size for each summary birth history estimate.

Summary birth history estimates are subject to both sampling error and model error, and we wanted to reflect this in our estimates of the effective number of births and deaths associated with each U5MR estimate derived from a summary birth history. To approximate the sampling variance of $U5M{R}_{j,t,s}^{(SBH)}$, we assumed the number of children that die is approximately binomially distributed:

$$ {\sigma}_{j,t,s\ \left[ sampling\right]}^2=\frac{U5M{R}_{j,t,s}^{(SBH)}\cdotp \left(1-U5M{R}_{j,t,s}^{(SBH)}\right)}{N_{j,t,s}^{\ast (SBH)}} $$

To approximate the model variance, we utilized the results of the validation exercise reported by Rajaratnam et al. [15] Five-fold cross-validation was used to assess the performance of the summary birth history methods in reproducing estimates derived from complete birth histories. We calculated the variance of the residuals from this comparison for each year prior to survey – i.e., the difference between the summary birth history estimate and corresponding complete birth history estimate, on a probability scale – as an approximation of the error introduced by using the summary birth history data and methods compared to the complete birth history data and methods. For each estimate of $U5M{R}_{j,t,s}^{(SBH)}$, we used this variance, matched for appropriate number of years prior to survey, as ${\sigma}_{j,t,s\ \left[ model\right]}^2$. We assumed that the model error and sample error were independent, and calculated the total variance for each estimate of $U5M{R}_{j,t,s}^{(SBH)}$ as

$$ {\sigma}_{j,t,s\ \left[ total\right]}^2={\sigma}_{j,t,s\ \left[ sampling\right]}^2+{\sigma}_{j,t,s\ \left[ model\right]}^2, $$

and calculated the corresponding effective sample size ${N}_{j,t,s}^{(SBH)}$ again assuming that the number of children who die is approximately binomially distributed:

$$ {N}_{j,t,s}^{(SBH)}=\frac{U5M{R}_{j,t,s}^{(SBH)}\cdotp \left(1-U5M{R}_{j,t,s}^{(SBH)}\right)}{\sigma_{j,t,s\ \left[ total\right]}^2} $$

This procedure was carried out both at the national level as well as each subnational area at the finest level available in given survey or census, and the resulting values of ${N}_{j,t,s}^{(SBH)}$ for all subnational areas were scaled to sum to the estimated value of ${N}_{j,t,s}^{(SBH)}$for the country as a whole. Finally, we calculated the effective number of deaths by multiplying the estimated U5MR by the effective number of births:

$$ {Y}_{j,t,s}^{(SBH)}=U5M{R}_{j,t,s}^{(SBH)}\cdotp {N}_{j,t,s}^{(SBH)} $$

### Small area models

We started with the following hierarchical generalized linear model defined for data stratified by area (department, district, or sub-district, depending on the country), year, and data source (a single survey or census in a particular area):

$$ {\displaystyle \begin{array}{c}{Y}_{j,t,s}\sim \mathrm{Binomial}\left({p}_{j,t,s},{N}_{j,t,s}\right)\\ {}\mathrm{logit}\left({p}_{j,t,s}\right)={\beta}_0+{u}_{0,j}+{\sum}_{i=1}^5\left({\beta}_i+{u}_{i,j}\right)\cdot {S}_i(t)+{\gamma}_s\end{array}} $$

where

*N*_*j*, *t*, *s*_ and *Y*_*j*, *t*, *s*_ are the number of births and deaths respectively in area *j*, year *t,* and source *s* (equivalent to ${N}_{j,t,s}^{(CBH)}$ and ${Y}_{j,t,s}^{(CBH)}$ or ${N}_{j,t,s}^{(SBH)}$ and ${Y}_{j,t,s}^{(SBH)}$, depending on the birth history method used to analyze source *s*);

*p*_*j*, *t*, *s*_ is the underlying U5MR in area *j*, year *t,* and source *s*;

*β*_0_ and *u*_0, *j*_ are the country-level fixed intercept and the area-level random intercept, respectively;

*S*_*i*_(*t*) is basis *i* of a natural cubic spline [16] with four equally-spaced interior knots evaluated at time *t*;

*β*_*i*_ and *u*_*i*, *j*_ are the country-level fixed slopes and area-level random slopes on *S*_*i*_(*t*), respectively;

and *γ*_*s*_ is a source-level random intercept.

We then added a second component which allows us to incorporate data defined for other geographic levels (i.e., higher level or historical administrative units):

$$ {\displaystyle \begin{array}{c}{Y}_{k,t,s}\sim \mathrm{Binomial}\left({p}_{k,t,s},{N}_{k,t,s}\right)\\ {}\mathrm{logit}\left({p}_{k,t,s}\right)=\mathrm{logit}\left({\sum}_{j\in k}\frac{P_{j,t}}{P_{k,t}}\cdot {p}_{j,t}\right)+{\gamma}_s=\mathrm{logit}\left({p}_{k,t}\right)+{\gamma}_s\end{array}} $$

Where *p*_*k*, *t*, *s*_, *N*_*k*, *t*, *s*_, *Y*_*k*, *t*, *s*_ are defined analogously to *p*_*j*, *t*, *s*_, *N*_*j*, *t*, *s*_, *Y*_*j*, *t*, *s*_ but for some area *k* made up of multiple area *j*’s (e.g., a province, containing multiple districts). *p*_*j*, *t*_, the underlying U5MR in area *j* and year *t* is defined analogously to *p*_*j*, *t*, *s*_ above, but with *γ*_*s*_ set to 0, and *p*_*k*, *t*_, the true prevalence in area *k* and year *t,* is given by the population (*P*) weighted average of *p*_*j*, *t*_ across all areas *j* contained within area *k*.

The random effect terms *u*_0, *j*_ and *u*_*i*, *j*_ (*i* = 1–5) were assigned conditional autoregressive priors as described by Leroux et al. [17] These priors allow for spatial smoothing based on the neighborhood structure of the areas being modeled, specifically by assuming that the prior mean for a given area is a function of the values in neighboring areas. The full conditional distribution implied by this prior is:

$$ {u}_j\mid {u}_{-j},{\sigma}^2,\rho \sim \mathrm{Normal}\left(\frac{\rho {\sum}_{m\sim j}{u}_m}{n_j\rho +1-\rho },\frac{\sigma^2}{n_j\rho +1-\rho}\right) $$

where *m*~*j* indicates that area *m* is a neighbor of (i.e., shares a boarder with) area *j* and *n*_*j*_ is the number of neighbors of area *j*. The two hyperparameters for each random effect were estimated as part of the model fitting process: *σ*^2^ determines the overall amount of variation and *ρ*, which varies between 0 and 1, determines the degree of spatial autocorrelation. Lower values of *ρ* indicate less spatial relatedness, while higher values of *ρ* indicate a high degree of spatial relatedness; at the extremes, this prior reduces to a Normal(0, 1) prior when *ρ* is 0 and to an intrinsic conditional autoregressive prior [18] where the prior mean is equal to the mean of all neighbors when *ρ* is 1. Normal(0, 10) hyper-priors were specified for logit-transformed *ρ* and half-Normal(0, 1) hyper-priors were specified for *σ* for all random effects. Common *σ*^2^ and *ρ* parameters were estimated for all random effects on the spline bases, *u*_*i*, *j*_ (*i* = 1–5). Posterior estimates of these hyperparameters are listed in Additional file 1.

Models were estimated separately for each country. All models were fit using the TMB package [19] in R version 3.2.4 [20]. We extracted point estimates and the variance-covariance matrix for logit(*p*_*j*, *t*_) and used these to generate 1000 draws of *p*_*j*, *t*_ by drawing from a multivariate normal distribution and then inverse-logit transforming each draw. These draws were scaled to match existing national-level estimates of U5MR from the Global Burden of Disease (GBD) Study: [3] for each year *t* and each draw, we calculated the ratio of the national estimate from the GBD to the national estimate derived from population-weighting *p*_*j*, *t*_ and multiplied *p*_*j*, *t*_ by this ratio. Finally, we calculated point estimates of *p*_*j*, *t*_ from the mean of these draws and the lower and upper bounds of the 95% uncertainty interval from the 2.5th and 97.5th percentiles, respectively. Relative change in *p*_*j*, *t*_ over time was also calculated for each draw, and 95% uncertainty intervals were derived from the 2.5th and 97.5th percentiles.

## Results

### Spatial patterns in U5MR

**Fig. 1:**
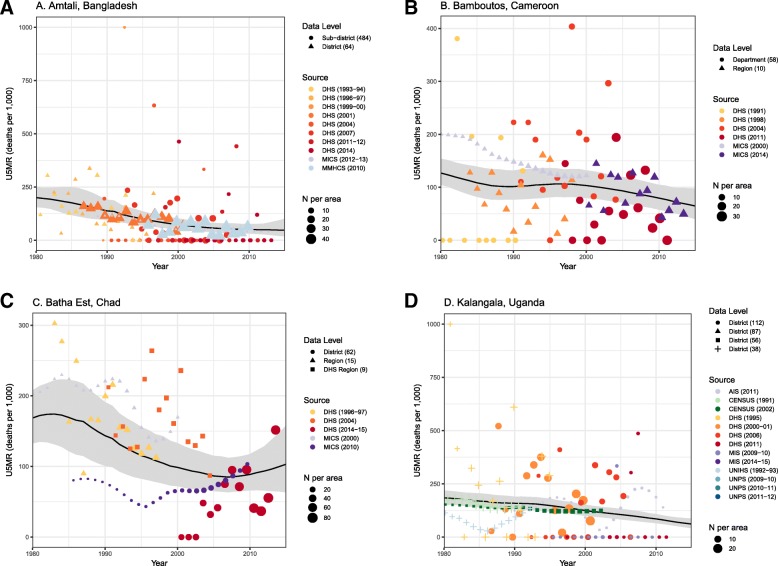
Data and estimates for selected areas. **a** Amtali, Bangladesh (sub-district); **b** Bamboutos, Cameroon (department); **c** Batha Est, Chad (department); **d** Kalangala, Uganda (district)

**Fig. 2:**
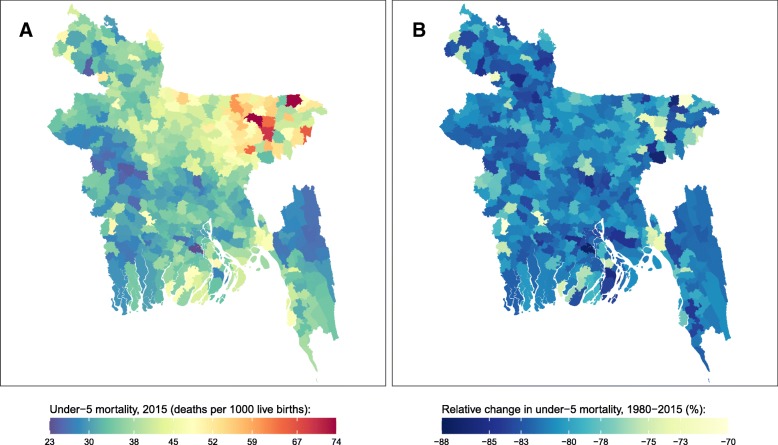
Under-5 mortality rate by sub-district in Bangladesh. **a** Under-5 mortality rate in 2015 and (**b**) relative change in the under-5 mortality rate between 1980 and 2015

**Fig. 3:**
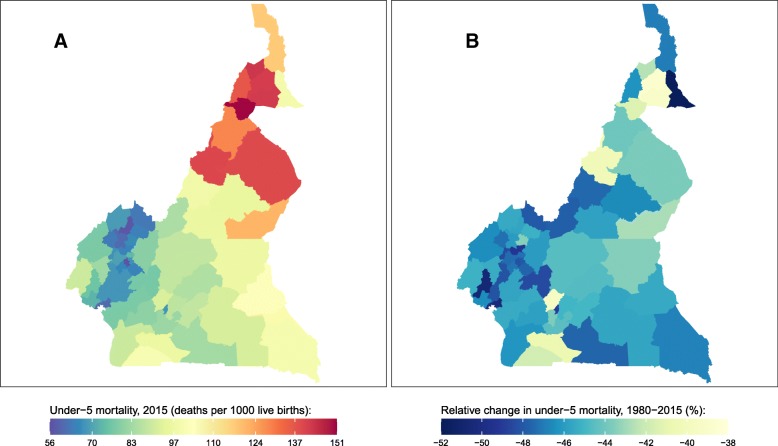
Under-5 mortality rate by department in Cameroon. **a** Under-5 mortality rate in 2015 and (**b**) relative change in the under-5 mortality rate between 1980 and 2015

**Fig. 4:**
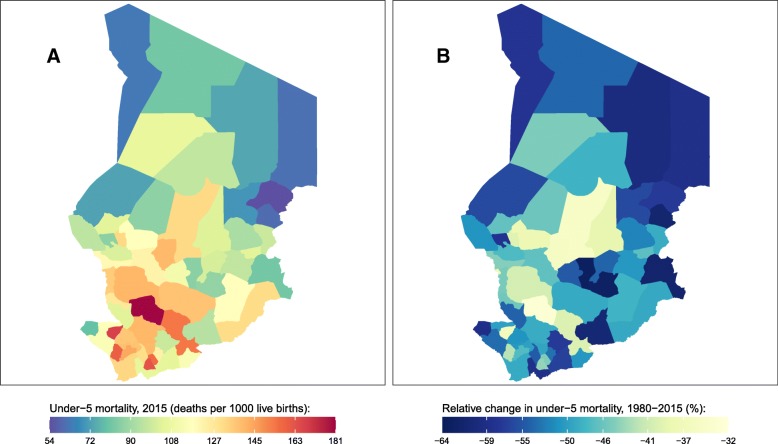
Under-5 mortality rate by department in Chad. **a** Under-5 mortality rate in 2015 and (**b**) relative change in the under-5 mortality rate between 1980 and 2015

**Fig. 5:**
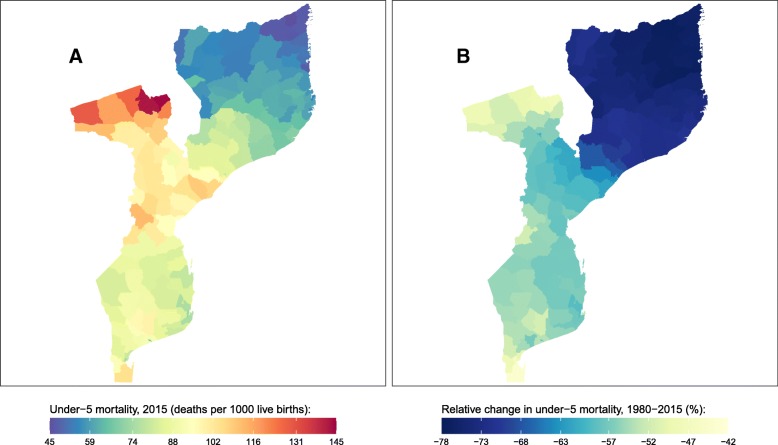
Under-5 mortality rate by district in Mozambique. **a** Under-5 mortality rate in 2015 and (**b**) relative change in the under-5 mortality rate between 1980 and 2015

**Fig. 6:**
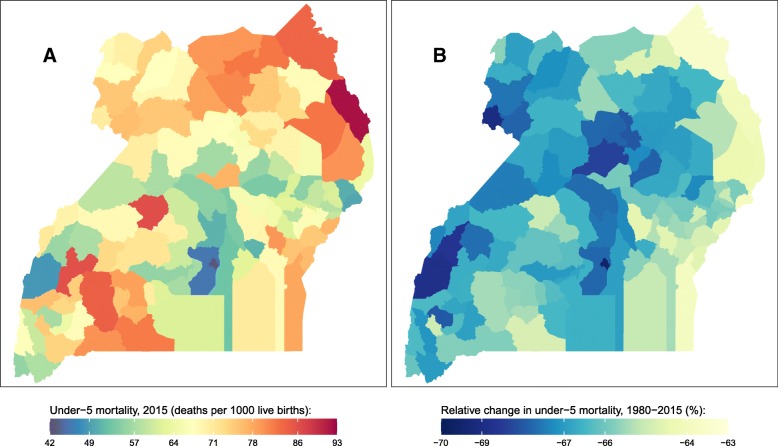
Under-5 mortality rate by district in Uganda. **a** Under-5 mortality rate in 2015 and (**b**) relative change in the under-5 mortality rate between 1980 and 2015

**Fig. 7:**
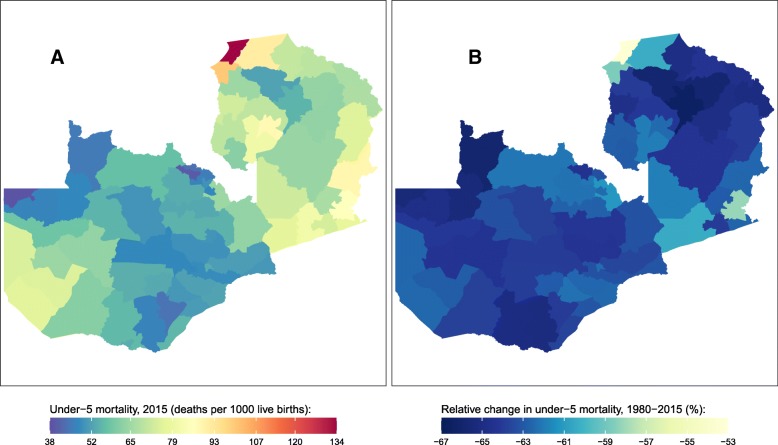
Under-5 mortality rate by district in Zambia. **a** Under-5 mortality rate in 2015 and (**b**) relative change in the under-5 mortality rate between 1980 and 2015

Figure 1 shows birth history and small area estimates for four example areas. Figures 2, 3, 4, 5, 6 and 7 show the predicted U5MR for each country in 2015 and the relative change from 1980 to 2015. Results for all years with associated uncertainty intervals are provided in Additional file 2. We found significant variation in U5MR at the second (or third, in Bangladesh) administrative level in all six countries.

In 2015, U5MR in Bangladesh was 36 (95% uncertainty interval: 33, 41) deaths per 1000 live births; however, U5MR varied among sub-districts from 23 (10, 44) in Barisal sub-district to 74 (32, 141) in Derai sub-district. In general, the highest U5MRs were found in northeastern sub-districts, while the lowest were found in eastern and central-western sub-districts. Between 1980 and 2015, U5MR improved in all sub-districts, with relative declines ranging from 70% (43, 87) in Jhikargachha sub-district to 87% (75, 94) in Barisal sub-district.

U5MR in Cameroon in 2015 was 91 (74, 112) deaths per 1000 live births. At the department level, there was a more than two-fold difference between U5MR in Hauts Plateauxand (57 [34, 87]) and Mayo Louti (150 [99, 218]). Overall, the highest mortality rates were found in northern departments while departments in the central and eastern parts of the country experienced more intermediate U5MRs, and departments in the northwest experienced the lowest U5MRs. U5MR declined in all departments in Cameroon between 1980 and 2015, but the magnitude of these declines varied widely, from 39% (12, 60) in Diamare to 52% (31, 69) in Mayo Danay.

Chad experienced an U5MR of 117 (103, 136) deaths per 1000 live births in 2015. At the department level, U5MR ranged from 54 (29, 91) in Kobe to 180 (121, 260) in Loug Chari. Departments in the southwest generally had the highest mortality rates while departments with relatively low mortality rates were concentrated in the northeast and northwest. Declines in U5MR between 1980 and 2015 ranged from 33% (8, 53) in Loug Chari to 63% (45, 76) in Guera.

U5MR in Mozambique was 81 (70, 93) deaths per 1000 live births in 2015. At the district level, U5MR varied from 45 (20, 94) in Cidade de Lichinga in Niassa province to 144 (79, 232) in Angonia in Tete province. In general, the lowest U5MRs were found in northern districts while the highest U5MRs were found in central-western districts. U5MR improved in all districts between 1980 and 2015, with declines ranging from 42% (− 27, 78) in Distrito Urbano 7 in Maputo to 77% (65, 86) in Mueda in Cabo Delgado province. There were distinct regional patterns in the decline in U5MR between 1980 and 2015, with the largest improvements found in northern districts and the smallest improvements found in central-western and southern districts.

In 2015, U5MR in Uganda was 66 (58, 75) deaths per 1000 live births; however, U5MR varied among districts from 42 (28, 62) in Kampala to 92 (60, 135) in Moroto. Large-scale spatial patterns in U5MR in 2015 were less prominent in Uganda compared to other countries. However, there were clusters of districts with higher than average U5MR in the northeast and southwest, and clusters of districts with lower than average U5MR in the central part of the country. Between 1980 and 2015, U5MR declined in all districts in Uganda. The rate of decline was more uniform in Uganda than the other countries considered, varying between 64% (48, 76) in Kaabong and 70% (57, 80) in Kampala.

Zambia experienced a U5MR of 62 (49, 76) deaths per 1000 in 2015. U5MR varied among districts by more than a factor of three, with the lowest rate found in Chavuma (39 [21, 66]) and the highest in Chiengi (134 [87, 190]). In general, lower mortality rates were found in the Copperbelt region and in more central districts, while higher mortality rates were found in districts in the east, north, and southwest. U5MR declined in all districts in Zambia between 1980 and 2015, with declines ranging from 53% (34, 68) in Chiengi to 66% (56, 76) in Kasama.

### Temporal and cross-country comparisons of the subnational distribution of U5MR

**Fig. 8:**
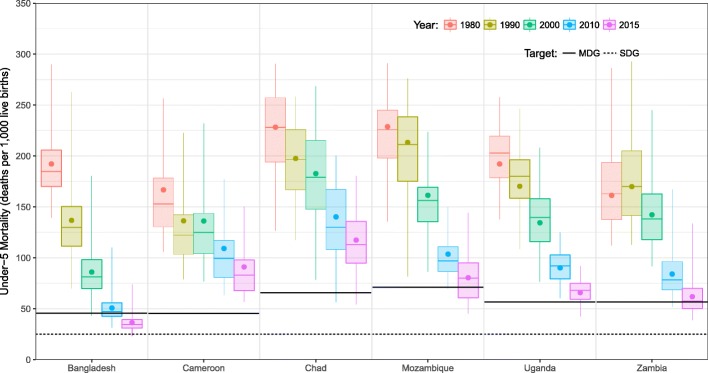
Subnational under-5 mortality rates compared to international targets, 1980–2015. Within each boxplot vertical lines indicate the range, boxes indicate the interquartile range, horizontal lines indicate the median, and dot indicates the national rate. The solid black lines indicate the country-level Millennium Development Goal target (i.e., a two-thirds reduction of the 1990 U5MR by 2015) and the black dashed line indicates the Sustainable Development Goal target (25 deaths per 1000 by 2030)

Figure 8 depicts how the distribution of U5MR at the second (or third) administrative level changed over time in each country. In Bangladesh, Chad, Mozambique, and Uganda, the distribution shifted downwards over the course of each decade in our analysis. In contrast, this distribution shifted upwards slightly in Cameroon between 1990 and 2000 and in Zambia between 1980 and 1990, though over the analysis period as a whole the same downwards shift was observed.

Among these six countries, only Bangladesh and Uganda have no overlap between the distribution of U5MR in 1980 and in 2015. In each of the remaining countries – Cameroon, Chad, Mozambique, and Zambia – there are areas where U5MR was higher in 2015 than in other areas of the same country three and half decades earlier. The comparison between 1980 and 2015 is particularly extreme in Cameroon, where the highest U5MR observed in any department in 2015 was similar to the median observed among departments in 1980.

Figure 8 shows the MDG target for each country as a solid black line. Among the six countries considered, only Bangladesh achieved the goal of a two-thirds reduction overall in U5MR between 1990 and 2015; most (87.2%), but not all, sub-districts in Bangladesh had U5MR below the country-wide MDG target in 2015 (based on the point estimates of U5MR in each area). In Chad, Mozambique, Uganda, and Zambia, a minority of areas reached the country-wide MDG target (6.5, 37.8, 16.1, and 48.6%, respectively). No department in Cameroon achieved the country-level MDG target in 2015.

Figure 8 also shows the Sustainable Development Goal (SDG) target for 2030 (25 deaths per 1000 for all countries). As of 2015, there was no district or department in Cameroon, Chad, Mozambique, Uganda, or Zambia where U5MR was below this threshold, while in Bangladesh four sub-districts had U5MR just below this threshold. Moreover, in all six countries there were areas where U5MR in 2015 was many times higher than the SDG target: e.g., in every country other than Bangladesh, a large majority (76.4–100%) of districts and departments had U5MR at least twice the SDG target.

### Comparison to alternate estimates

A comparison of U5MR estimates from this analysis to those in Golding et al. [11] is shown in Additional file 3. The point estimates from these two analyses are reasonably consistent for most countries in most years. However, there are important differences, particularly in Chad and Mozambique in 2015. The estimates from both analyses are associated with considerable uncertainty as indicated by wide uncertainty intervals; in nearly all cases (95.5% of area-years) these uncertainty intervals overlap between the two analyses.

## Discussion

Previous analyses of U5MR for small subnational areas (e.g., the second or third administrative level) are rare, largely due to the statistical challenge of estimating rates based on small sample sizes. The few analyses of this nature that do exist have generally attempted to overcome the challenge posed by small numbers by utilizing small area estimation techniques and where possible, incorporating multiple data sources. However, these small area estimation methods have not been able to handle spatially misaligned data; for example, data where the geographies identified were more aggregate (e.g., first instead of second administrative level) or referred to historical administrative units that have since split. In this analysis, we described methods that built on these previous analyses but are capable of incorporating spatially misaligned data, increasing the amount of data available for analysis. We applied these methods to six countries to demonstrate the utility of this methodology and the results it produces. Among the six countries we considered, between 38% (Uganda) and 60% (Chad) of the available data sources did not include sufficient geographic information to identify the areas of interest. We were nonetheless able to incorporate all of these data, making estimation of time trends at the second (or third, in Bangladesh) administrative level possible.

These estimates exposed a high degree of spatial heterogeneity in all six countries. In 2015, there was more (sometimes much more) than a two-fold difference between the highest and lowest U5MR found in each country. The relative change in U5MR over the past three and half decades was also highly variable in most countries, highlighting within-country differences in trajectory in addition to level. As countries consider how to reduce U5MR, these types of geographically precise estimates can be used to identify areas where attention and resources are most needed, or to highlight areas that have done well and might be mined for effective strategies.

These results also suggest that subnational estimates should be considered when tracking progress towards the SDGs. The MDGs and SDGs have increased attention paid to improving child survival; however, focusing on country-level metrics when evaluating progress towards these targets may mean that the benefits of this extra attention are not felt everywhere. Indeed, it is possible for improvements overall to be accompanied by increasing rather than decreasing inequalities [21]. Subnational estimates can be used to proactively identify areas where achieving the SDG target will take the most work but also the areas where there is the most room for improvement. Paying special attention to these areas will help ensure that they get the help they need while also contributing to improvements for the country as a whole.

There are likely many factors contributing to the spatial and temporal trends in U5MR described in this study. While attributing these trends to specific drivers is outside the scope of this analysis, factors such as poverty, urbanicity, malaria endemicity, and conflict and armed violence likely play an important role both in determining country-wide temporal trends in U5MR as well as regional differences within each country. Moreover, during the time period considered, all six countries have instituted numerous programs and policies to specifically combat common causes of child death and more generally expand and strengthen the health system. For example, in Bangladesh, the Expanded Program on Immunization (EPI) launched in 1979 led to a rapid increase in childhood vaccination rates; Bangladesh later introduced the Integrated Management of Childhood Illness (IMCI) program in 2002 to combat major causes of child mortality [22]. In Cameroon, 2010 updates to the Health Sector Strategy established programs to combat endemic diseases like malaria; improve reproductive health; expand immunization coverage via the EPI; and promote development of human resources, infrastructure, and technology within the health system. In Chad, increased oil revenues since 2003 have allowed the government to significantly increase spending on health programs and the number of health facilities and personnel. In Mozambique, the government and international response to the HIV crisis in the late 1990s has ultimately resulted in expanded access to HIV prevention and treatment services and buttressed the health system to deliver general health services [23]. Uganda has implemented a series of policies and programs targeting common causes of child death, including adopting IMCI guidelines in the late 1990s; introducing pentavalent vaccine in 2002; introducing Artemisinin Combination Therapy (ACTs) as the first line treatment for malaria in 2004; introducing pneumococcal vaccine in 2012; and mass distribution of insecticide treated nets (ITNs) in 2013 and 2014. Zambia similarly began implementing IMCI guidelines in some districts in 1996; adopted ACTs as the first-line treatment for malaria in 2004; and introduced the pentavalent vaccine in 2005 and the pneumococcal and rotavirus vaccines in 2013. However, in all six countries, the benefits of these various policies and programs have likely not been evenly distributed geographically [9, 24, 25].

### Limitations

This analysis is subject to a number of limitations. First, the birth history data we used as the primary source of information on U5MR are subject to misreporting and survival biases [26, 27]. Previous research has suggested that the impact of survival bias can be significant in populations with high HIV prevalence. Several methods have been proposed for correcting for this bias, and additional research is warranted to investigate integrating these methods alongside small area estimation methodologies [28–30]. Second, surveys that include GPS coordinates generally have these coordinates displaced to some degree to protect respondent’s confidentiality (for example, Demographic and Health Surveys randomly displace GPS coordinates up to 1 km in urban areas, up to 5 km in rural areas, and up to 10 km in a random 1% subset of survey clusters [31]). This displacement is not accounted for when mapping GPS data to administrative boundaries, potentially leading to some misclassification in areas near administrative borders. Third, the population data which we used to inform how different levels of geographic aggregation relate to each other in the small area estimation models are also subject to error, both due to errors in the underlying census data (e.g., omissions or age misreporting) as well as potential violation of the assumptions used to interpolate these data (e.g., due to migration). Fourth, the methods used to account for both sampling and model error and estimate the effective number of births associated with U5MR estimates from summary birth histories require a number of assumptions and approximations that may not be appropriate in all cases. Fifth, the small area estimation models smooth over space and time by making assumptions about the temporal and spatial structure of U5MR that may not always hold. Sixth, the small area models assume that the estimated deaths from the birth history data are binomially distributed for all areas, and this assumption may not be valid in all cases. Future research, potentially including simulation studies, should explore the implications of these assumptions as well as possible alternatives. Seventh, while the methods developed here allow for including spatially misaligned data, they do require that this spatial misalignment is of a particular type: specifically, that all larger areas are composed of simple combinations of the smaller areas of interest. Eighth, the estimates we derive are uncertain, as reflected by the uncertainty intervals that are often quite wide. Given this uncertainty, the results must be interpreted with caution. Ninth, we make a number of cross-country comparisons of the distribution of U5MR within each country, but this distribution is likely sensitive to how a given country is partitioned (i.e., the modifiable areal unit problem) [32]. Finally, it is difficult to assess the accuracy of these estimates, which may be compromised due either to violations of the modeling assumptions or quality issues in the underlying data sources. The estimates from this study generally have high face-validity given existing knowledge of the social and health context of each country. However, there are exceptions. In particular, estimates for recent years in Mozambique are difficult to explain, as some districts with relatively high and others with relatively low estimated U5MR are similar with regards to human and financial resource inputs (e.g., Macanga and Nangande). Future research should focus on formally validating these methods and comparing them to available alternatives.

## Conclusions

The methods described in this analysis provide a framework for efficiently utilizing all available data sources to estimate U5MR at a fine geographic level. Subnational estimates of U5MR based on this methodology reveal significant within-country variation. These estimates could be used for identifying high-need areas, tracking trends in geographic inequalities, and evaluating progress towards international development targets such as the Sustainable Development Goals.

## Additional files

Additional file 1: Posterior inference for hyperparameters σ and ρ. (DOCX 16 kb)

Additional file 2: Under-5 mortality estimates with uncertainty. (XLSX 796 kb)

Additional file 3: Comparison of U5MR estimates in this analysis and Golding et al. (PDF 84 kb)
