# Supplementary material for: Small area estimation of under-5 mortality in Bangladesh, Cameroon, Chad, Mozambique, Uganda, and Zambia using spatially misaligned data
Source: Popul Health Metr. 2018 Aug 13;16:13. doi: 10.1186/s12963-018-0171-7 (PMC6090708; doi:10.1186/s12963-018-0171-7)
Supplement: Supplementary file 1 — Posterior inference for hyperparameters σ and ρ. (DOCX 16 kb) [file 12963_2018_171_MOESM1_ESM.docx]

Small area estimation of under-5 mortality in Bangladesh, Cameroon, Chad, Mozambique, Uganda, and Zambia using spatially misaligned data.

**Additional file 1:** Posterior inference for hyperparameters *σ* and *ρ*, by country.

| Country | $u_{0}$ | | $u_{1-5}$ | | $\gamma_{s}$ |
| --- | --- | --- | --- | --- | --- |
|  | $\sigma$ | $\rho$ | $\sigma$ | $\rho$ | $\sigma$ |
| Bangladesh | 0.262 (0.113) | 0.987 (0.261) | 0.471 (1.203) | 0.058 (0.049) | 0.155 (0.056) |
| Cameroon | 0.320 (0.218) | 0.959 (0.349) | 0.182 (1.367) | 0.006 (0.078) | 0.166 (0.089) |
| Chad | 0.372 (0.184) | 0.920 (0.388) | 0.377 (1.448) | 0.369 (0.096) | 0.196 (0.111) |
| Mozambique | 0.232 (0.159) | 0.977 (0.301) | 0.198 (1.284) | 0.979 (0.143) | 0.222 (0.157) |
| Uganda | 0.318 (0.056) | 0.542 (0.299) | 0.266 (1.022) | 0.614 (0.059) | 0.202 (0.067) |
| Zambia | 0.310 (0.140) | 0.928 (0.233) | 0.166 (1.262) | 0.696 (0.055) | 0.124 (0.066) |

*Mean (standard error)*
