# Supplementary material for: Small area estimation of under-5 mortality in Bangladesh, Cameroon, Chad, Mozambique, Uganda, and Zambia using spatially misaligned data
Source: Popul Health Metr. 2018 Aug 13;16:13. doi: 10.1186/s12963-018-0171-7 (PMC6090708; doi:10.1186/s12963-018-0171-7)

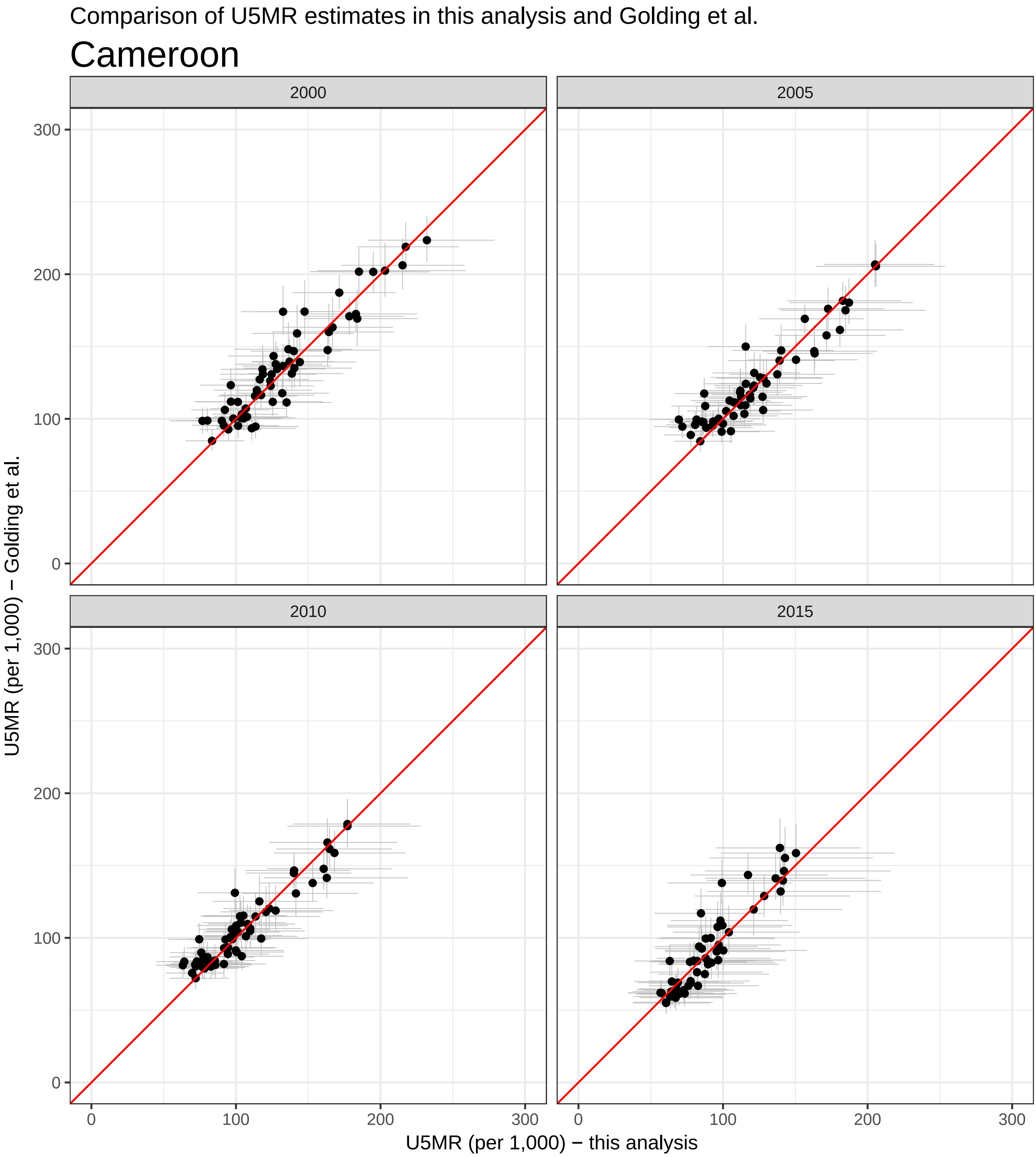
Comparison of U5MR estimates in this analysis and Golding et al.
Cameroon
2000
2005
2010
2015
U5MR (per 1,000) − Golding et al.
U5MR (per 1,000) − this analysis
0
100
200
300

Comparison of U5MR estimates in this analysis and Golding et al.

Chad

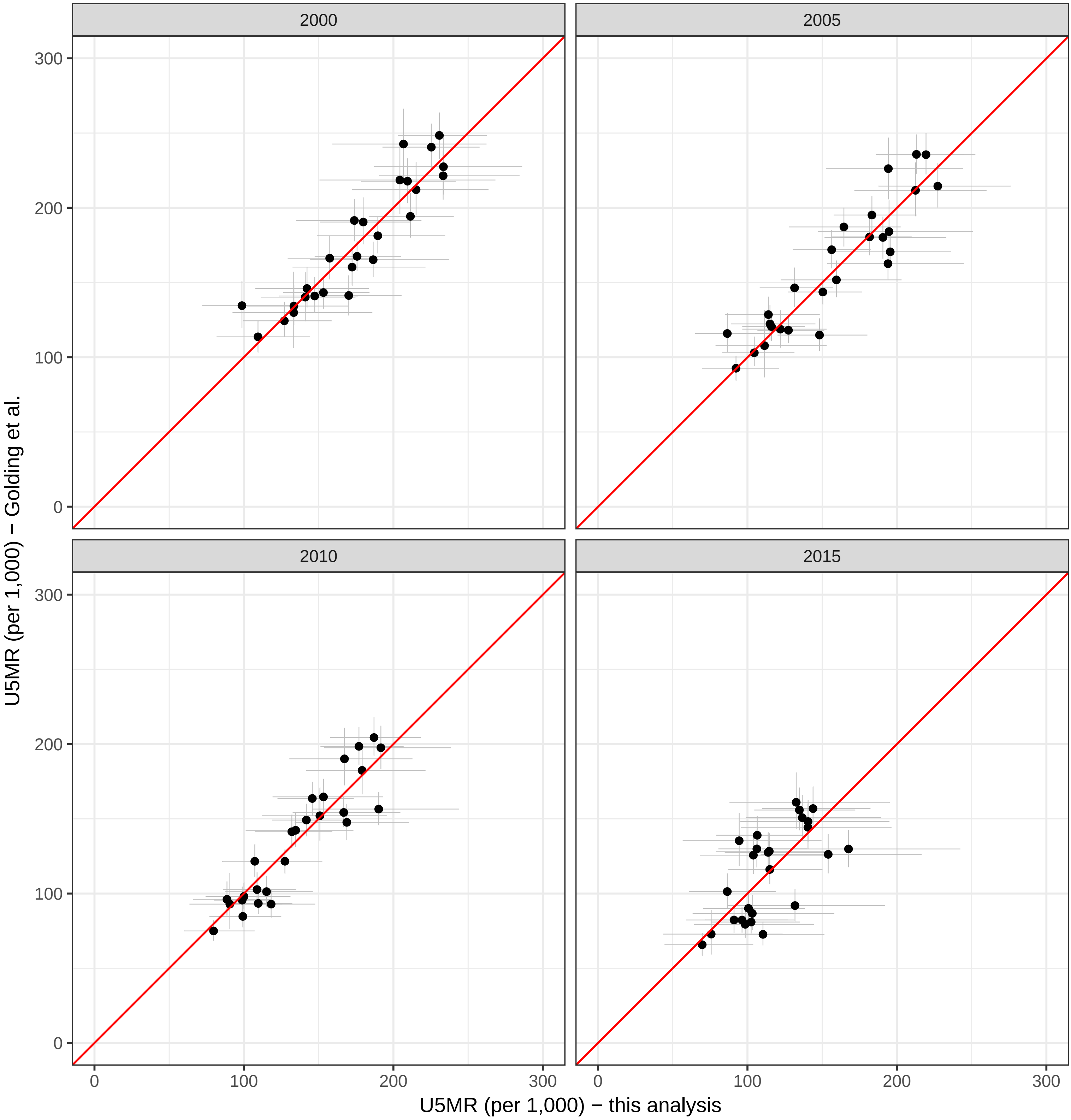

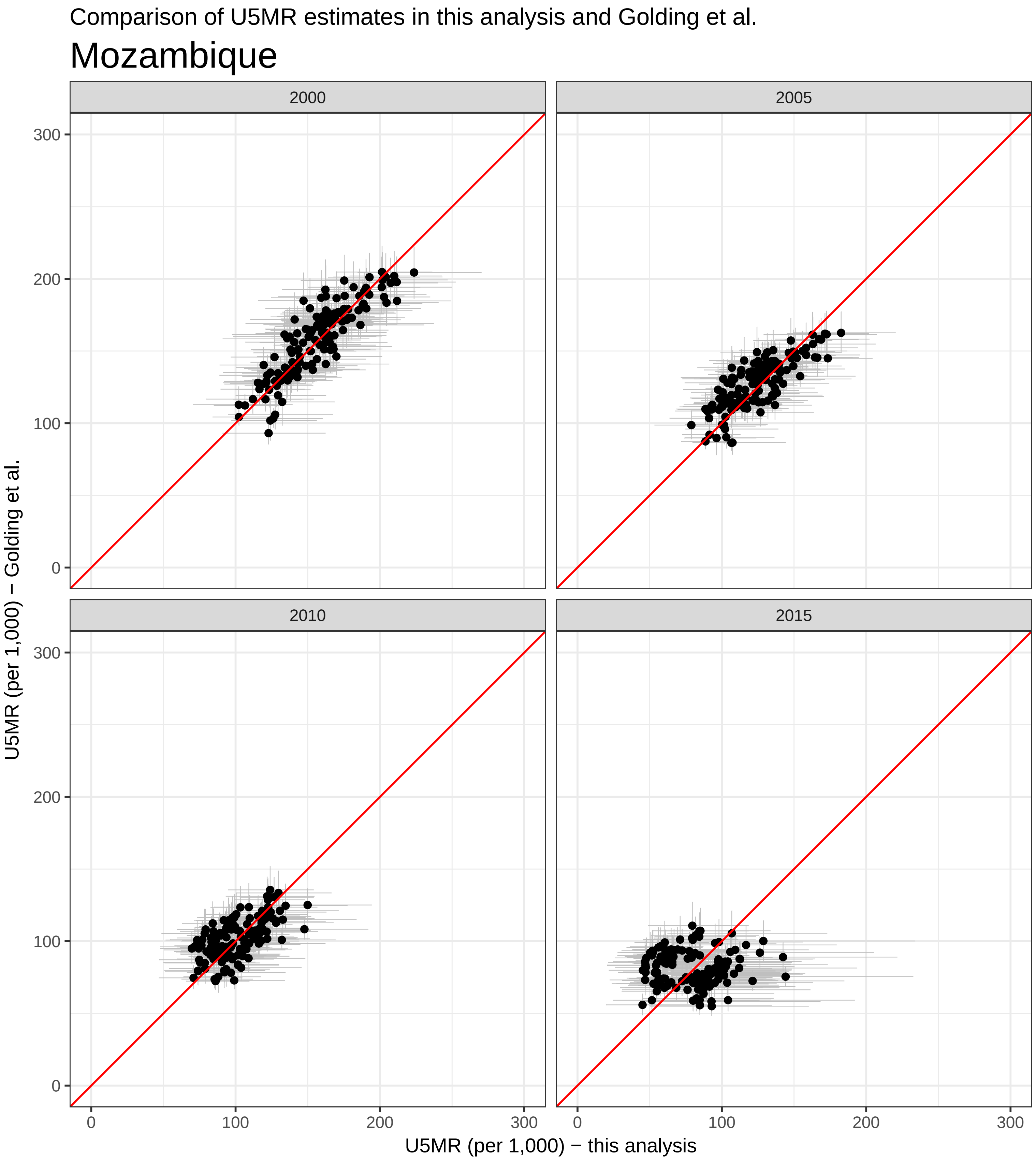
Comparison of U5MR estimates in this analysis and Golding et al.
Mozambique
2000
2005
2010
2015
U5MR (per 1,000) − Golding et al.
U5MR (per 1,000) − this analysis
0
100
200
300

Comparison of U5MR estimates in this analysis and Golding et al.

Uganda

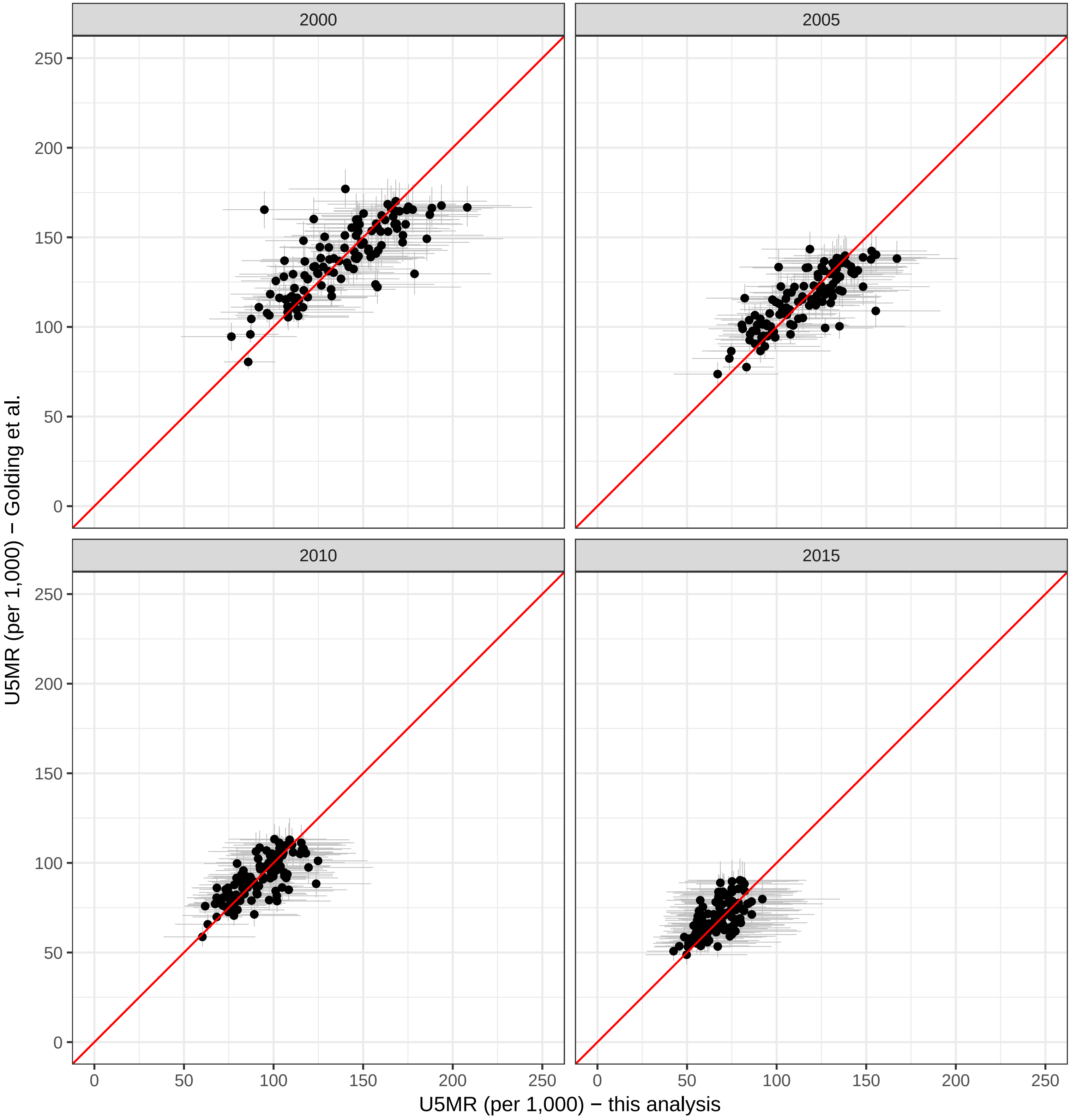

Comparison of U5MR estimates in this analysis and Golding et al.

Zambia

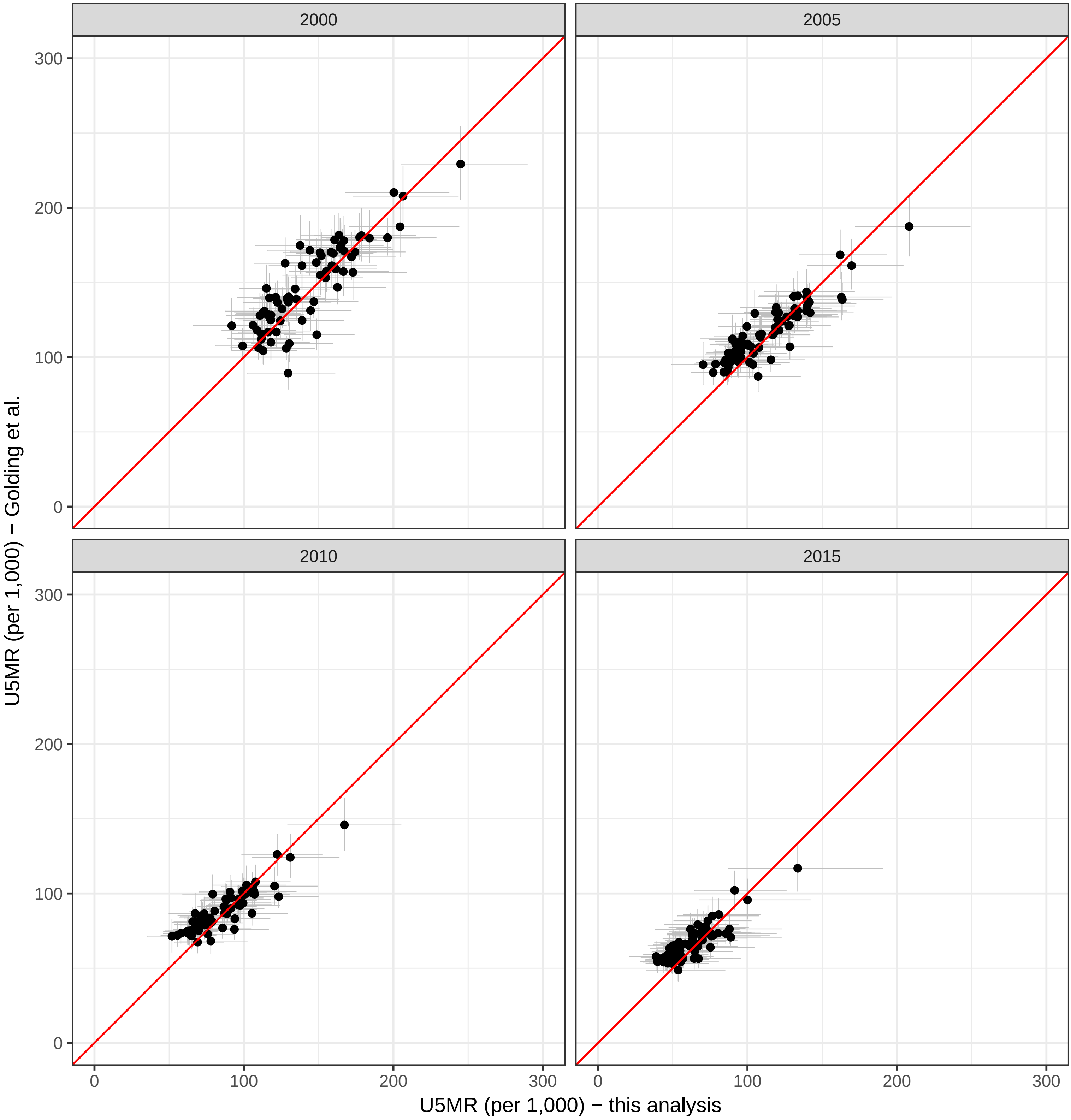

Supplement: Supplementary file 3 — Comparison of U5MR estimates in this analysis and Golding et al. (PDF 84 kb) [file 12963_2018_171_MOESM3_ESM.pdf]
